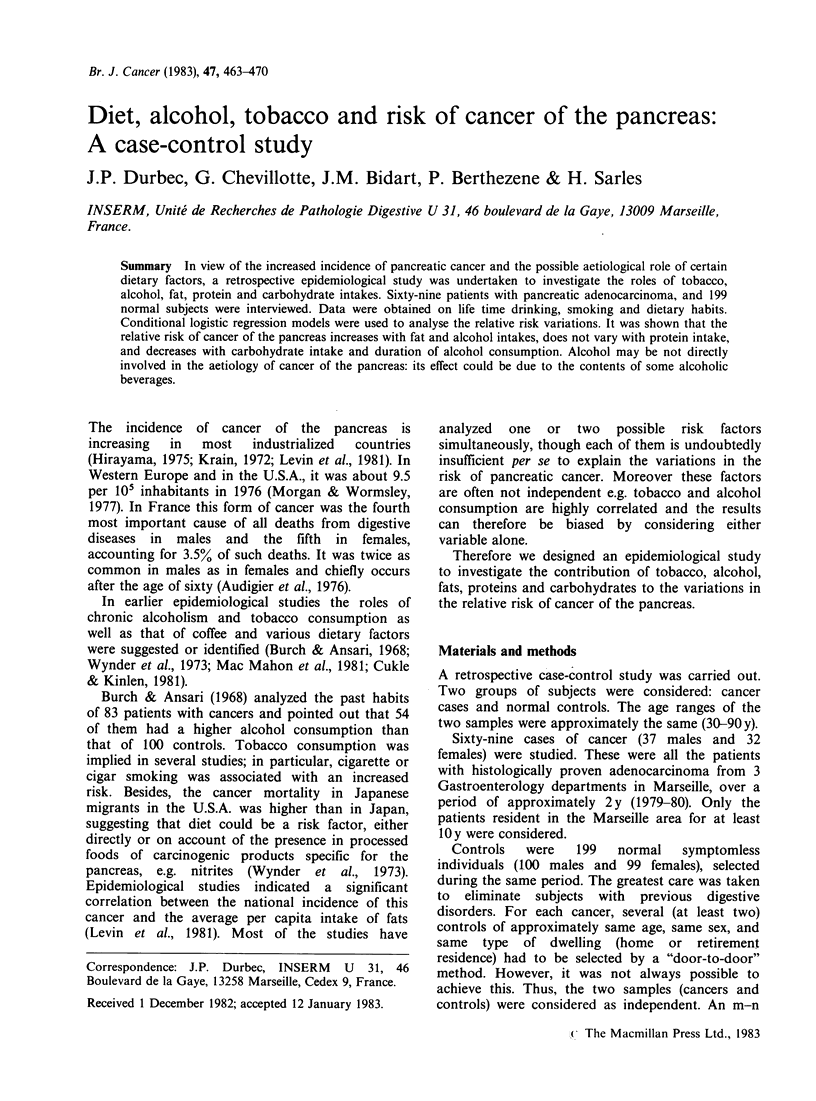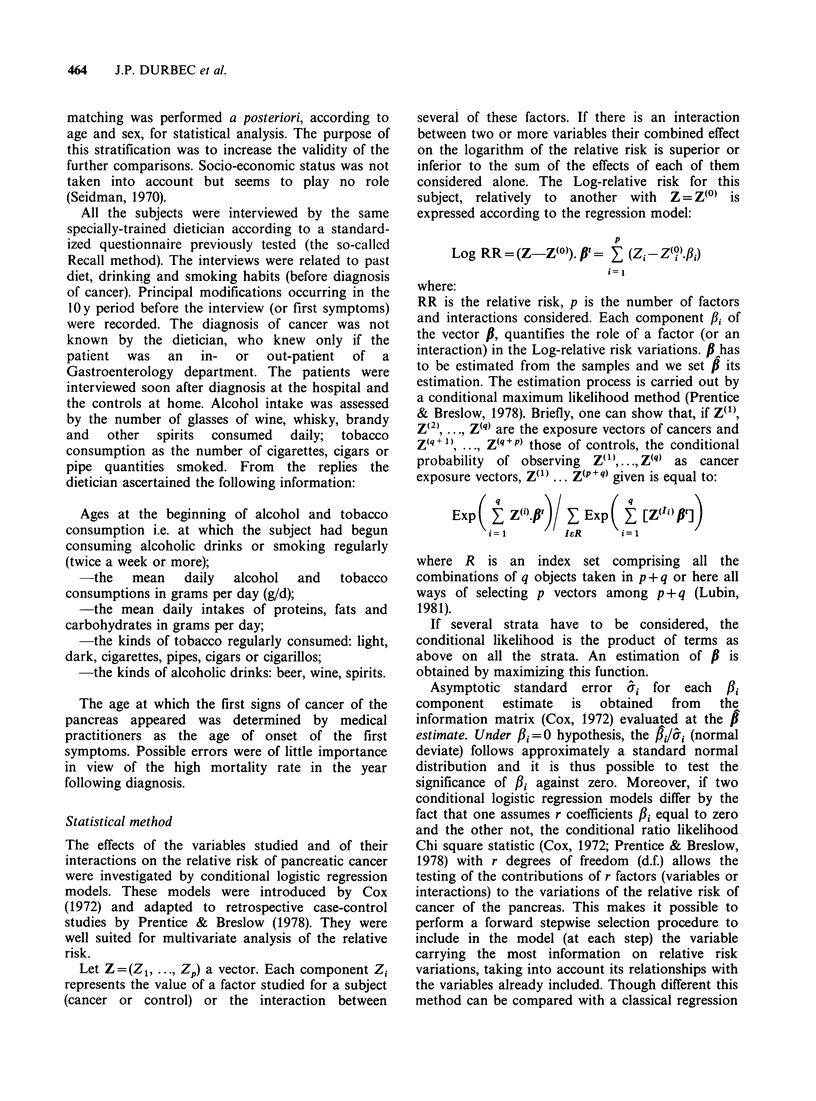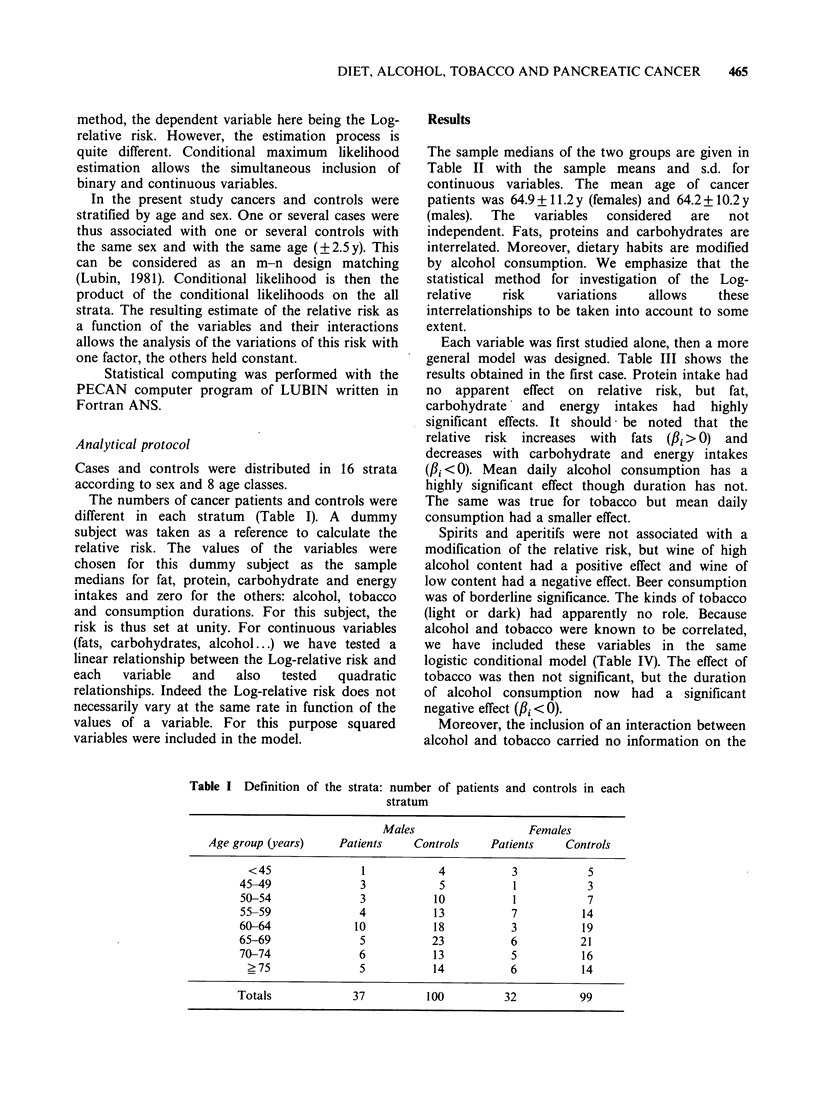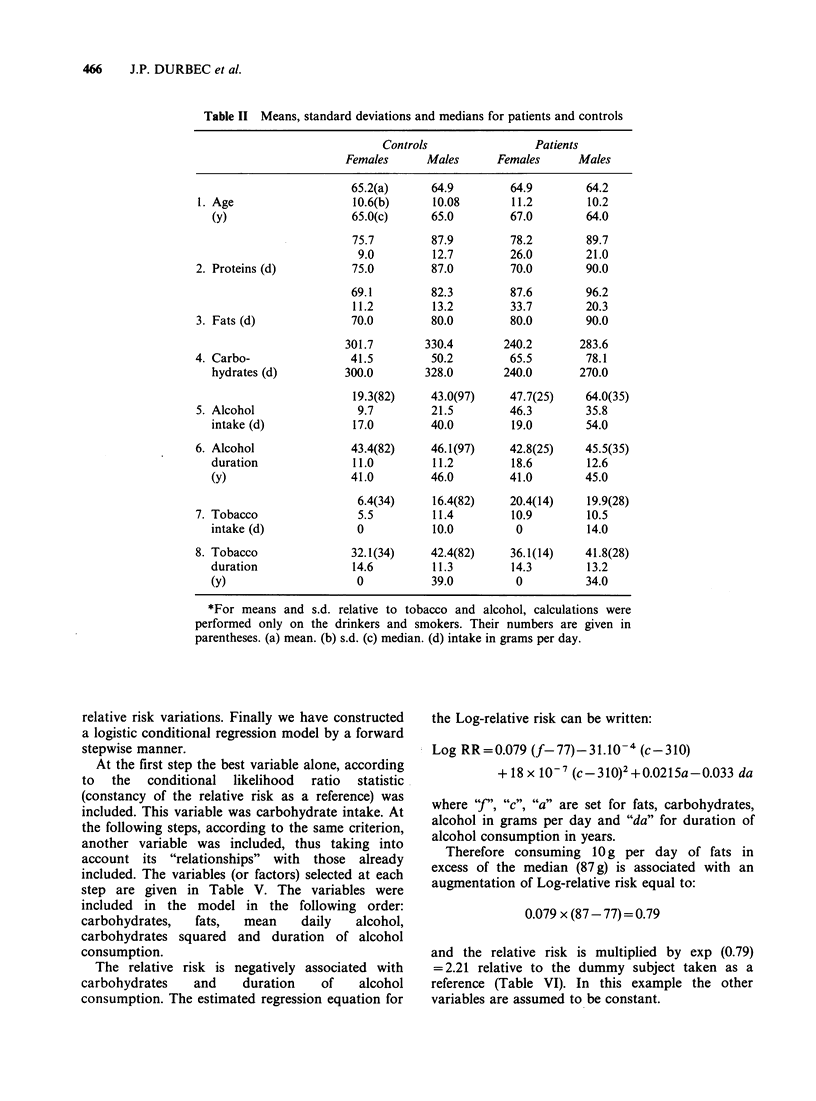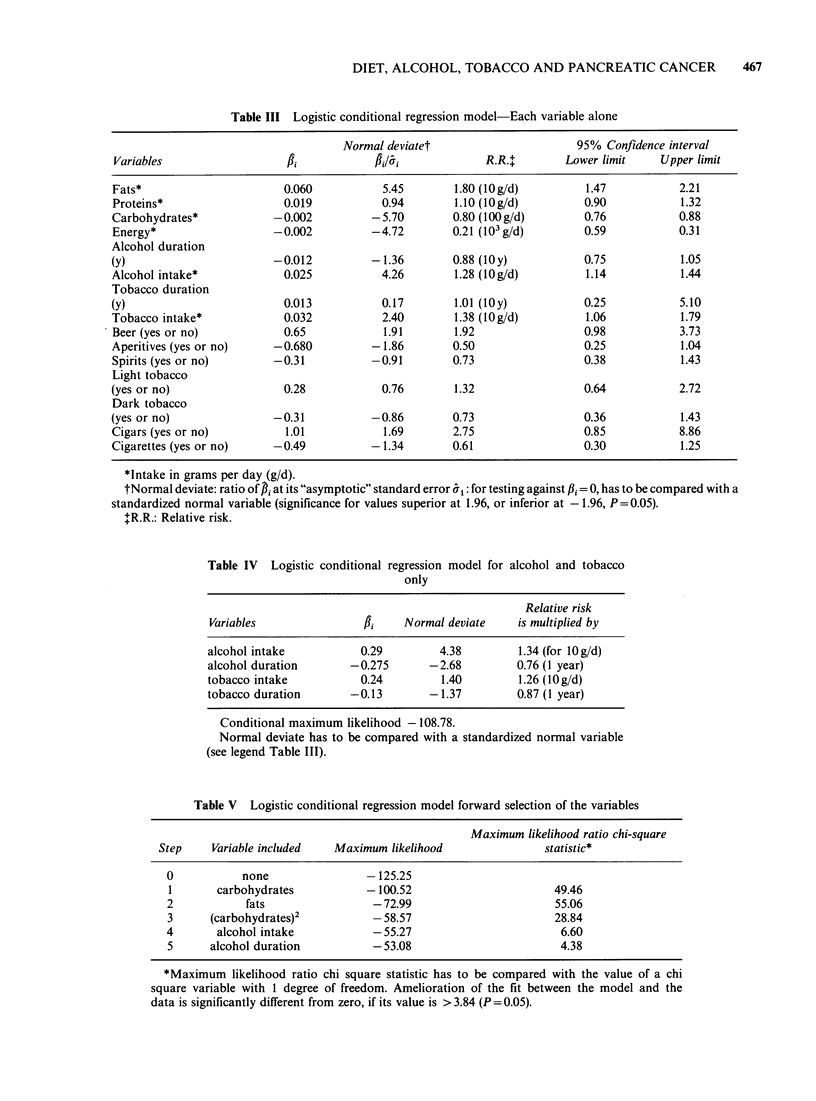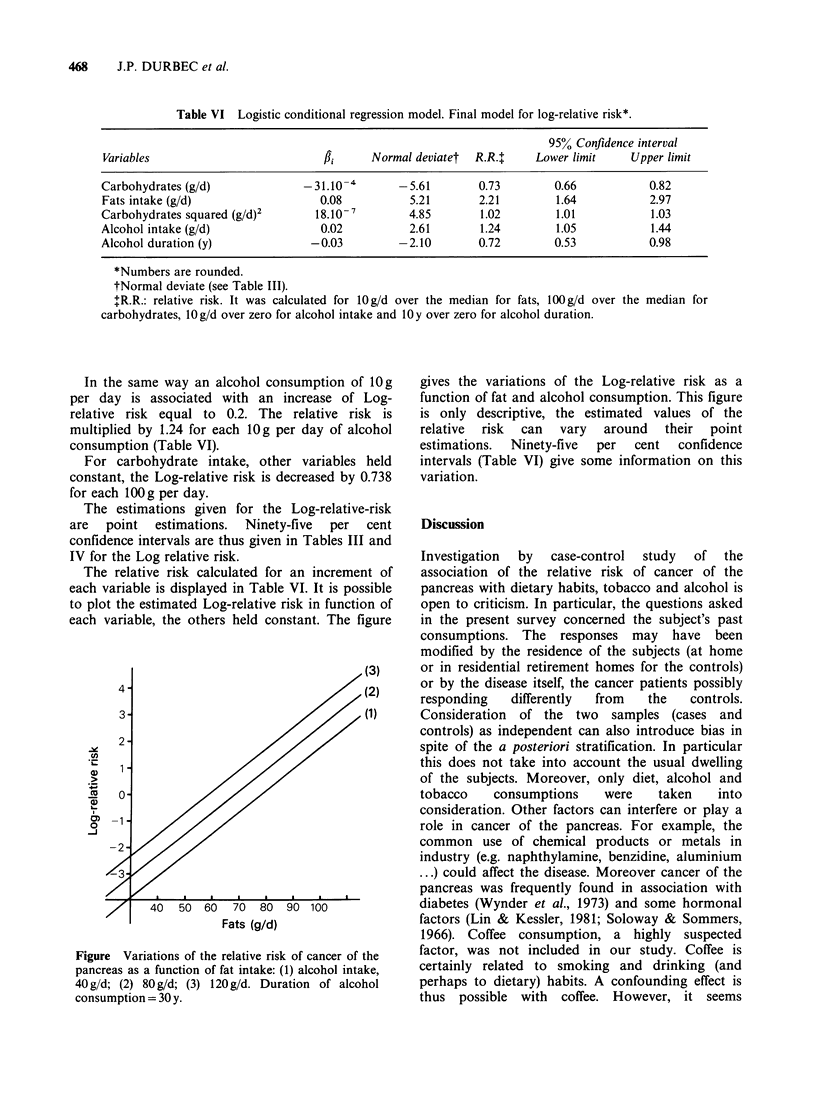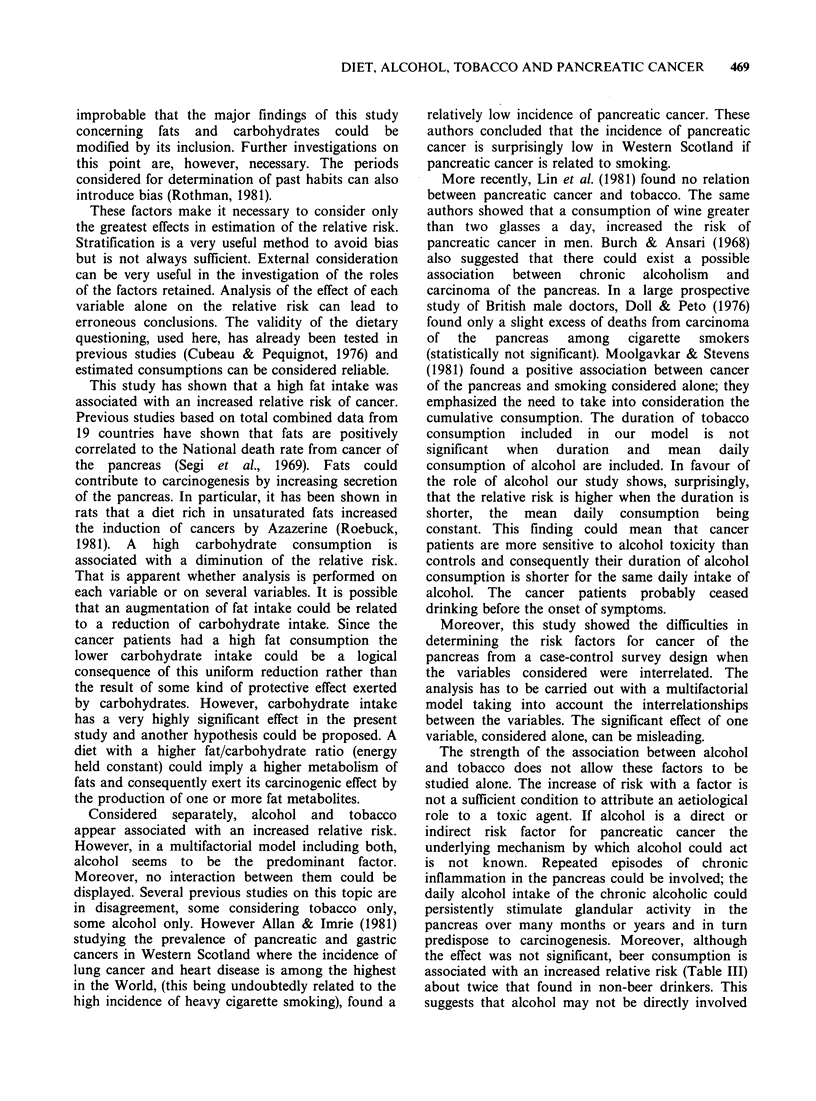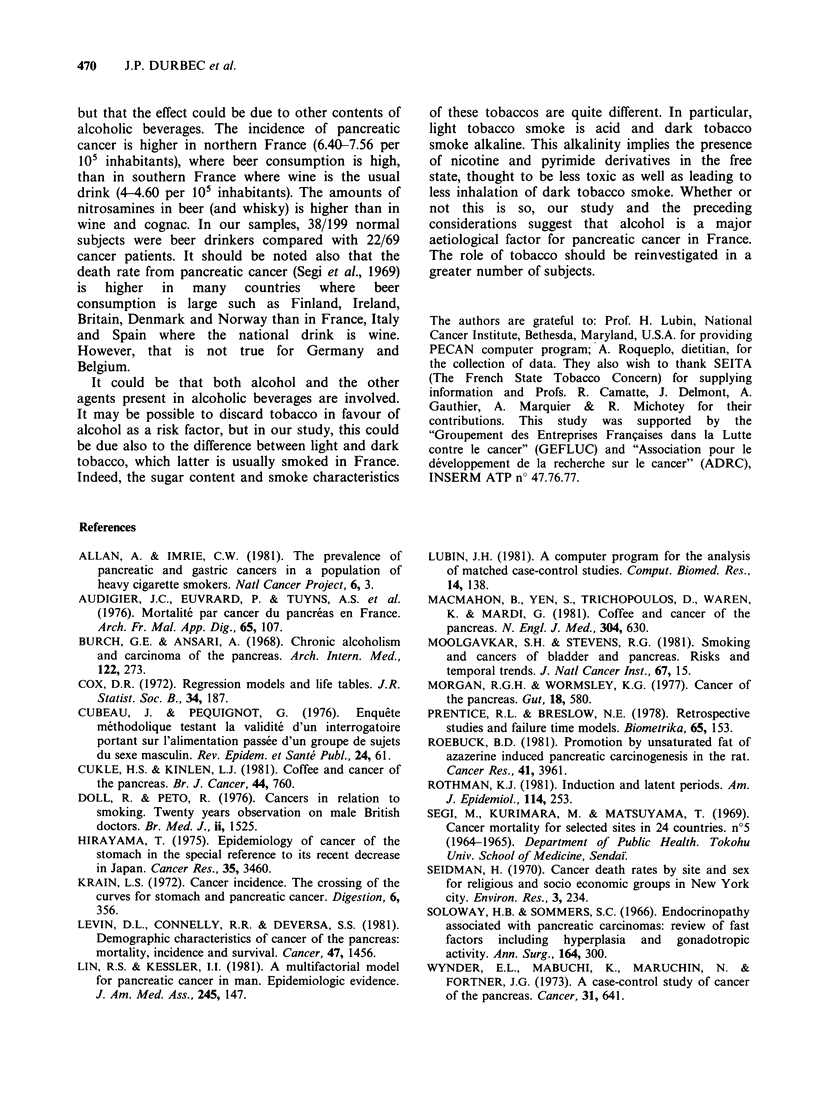# Diet, alcohol, tobacco and risk of cancer of the pancreas: a case-control study.

**DOI:** 10.1038/bjc.1983.75

**Published:** 1983-04

**Authors:** J. P. Durbec, G. Chevillotte, J. M. Bidart, P. Berthezene, H. Sarles

## Abstract

In view of the increased incidence of pancreatic cancer and the possible aetiological role of certain dietary factors, a retrospective epidemiological study was undertaken to investigate the roles of tobacco, alcohol, fat, protein and carbohydrate intakes. Sixty-nine patients with pancreatic adenocarcinoma, and 199 normal subjects were interviewed. Data were obtained on life time drinking, smoking and dietary habits. Conditional logistic regression models were used to analyse the relative risk variations. It was shown that the relative risk of cancer of the pancreas increases with fat and alcohol intakes, does not vary with protein intake, and decreases with carbohydrate intake and duration of alcohol consumption. Alcohol may be not directly involved in the aetiology of cancer of the pancreas: its effect could be due to the contents of some alcoholic beverages.


*Br. J. Cancer* (1983), **47**, 463–470

### Diet, alcohol, tobacco and risk of cancer of the pancreas: A case-control study

J.P. Durbec, G. Chevillotte, J.M. Bidart, P. Berthezene & H. Sarles

*INSERM, Unité de Recherches de Pathologie Digestive U 31, 46 boulevard de la Gaye, 13009 Marseille, France.*

**Summary** In view of the increased incidence of pancreatic cancer and the possible aetiological role of certain dietary factors, a retrospective epidemiological study was undertaken to investigate the roles of tobacco, alcohol, fat, protein and carbohydrate intakes. Sixty-nine patients with pancreatic adenocarcinoma, and 199 normal subjects were interviewed. Data were obtained on life time drinking, smoking and dietary habits. Conditional logistic regression models were used to analyse the relative risk variations. It was shown that the relative risk of cancer of the pancreas increases with fat and alcohol intakes, does not vary with protein intake, and decreases with carbohydrate intake and duration of alcohol consumption. Alcohol may be not directly involved in the aetiology of cancer of the pancreas: its effect could be due to the contents of some alcoholic beverages.

The incidence of cancer of the pancreas is increasing in most industrialized countries (Hirayama, 1975; Krain, 1972; Levin *et al.*, 1981). In Western Europe and in the U.S.A., it was about 9.5 per $10^5$ inhabitants in 1976 (Morgan & Wormsley, 1977). In France this form of cancer was the fourth most important cause of all deaths from digestive diseases in males and the fifth in females, accounting for 3.5% of such deaths. It was twice as common in males as in females and chiefly occurs after the age of sixty (Audigier *et al.*, 1976).

In earlier epidemiological studies the roles of chronic alcoholism and tobacco consumption as well as that of coffee and various dietary factors were suggested or identified (Burch & Ansari, 1968; Wynder *et al.*, 1973; Mac Mahon *et al.*, 1981; Cukle & Kinlen, 1981).

Burch & Ansari (1968) analyzed the past habits of 83 patients with cancers and pointed out that 54 of them had a higher alcohol consumption than that of 100 controls. Tobacco consumption was implied in several studies; in particular, cigarette or cigar smoking was associated with an increased risk. Besides, the cancer mortality in Japanese migrants in the U.S.A. was higher than in Japan, suggesting that diet could be a risk factor, either directly or on account of the presence in processed foods of carcinogenic products specific for the pancreas, e.g. nitrites (Wynder *et al.*, 1973). Epidemiological studies indicated a significant correlation between the national incidence of this cancer and the average per capita intake of fats (Levin *et al.*, 1981). Most of the studies have analyzed one or two possible risk factors simultaneously, though each of them is undoubtedly insufficient *per se* to explain the variations in the risk of pancreatic cancer. Moreover these factors are often not independent e.g. tobacco and alcohol consumption are highly correlated and the results can therefore be biased by considering either variable alone.

Therefore we designed an epidemiological study to investigate the contribution of tobacco, alcohol, fats, proteins and carbohydrates to the variations in the relative risk of cancer of the pancreas.

### Materials and methods

A retrospective case-control study was carried out. Two groups of subjects were considered: cancer cases and normal controls. The age ranges of the two samples were approximately the same (30–90 y).

Sixty-nine cases of cancer (37 males and 32 females) were studied. These were all the patients with histologically proven adenocarcinoma from 3 Gastroenterology departments in Marseille, over a period of approximately 2 y (1979–80). Only the patients resident in the Marseille area for at least 10 y were considered.

Controls were 199 normal symptomless individuals (100 males and 99 females), selected during the same period. The greatest care was taken to eliminate subjects with previous digestive disorders. For each cancer, several (at least two) controls of approximately same age, same sex, and same type of dwelling (home or retirement residence) had to be selected by a "door-to-door" method. However, it was not always possible to achieve this. Thus, the two samples (cancers and controls) were considered as independent. An m–n

Correspondence: J.P. Durbec, INSERM U 31, 46 Boulevard de la Gaye, 13258 Marseille, Cedex 9, France.

Received 1 December 1982; accepted 12 January 1983.

© The Macmillan Press Ltd., 1983

matching was performed *a posteriori*, according to age and sex, for statistical analysis. The purpose of this stratification was to increase the validity of the further comparisons. Socio-economic status was not taken into account but seems to play no role (Seidman, 1970).

All the subjects were interviewed by the same specially-trained dietician according to a standardized questionnaire previously tested (the so-called Recall method). The interviews were related to past diet, drinking and smoking habits (before diagnosis of cancer). Principal modifications occurring in the 10 y period before the interview (or first symptoms) were recorded. The diagnosis of cancer was not known by the dietician, who knew only if the patient was an in- or out-patient of a Gastroenterology department. The patients were interviewed soon after diagnosis at the hospital and the controls at home. Alcohol intake was assessed by the number of glasses of wine, whisky, brandy and other spirits consumed daily; tobacco consumption as the number of cigarettes, cigars or pipe quantities smoked. From the replies the dietician ascertained the following information:

Ages at the beginning of alcohol and tobacco consumption i.e. at which the subject had begun consuming alcoholic drinks or smoking regularly (twice a week or more);

—the mean daily alcohol and tobacco consumptions in grams per day (g/d);

—the mean daily intakes of proteins, fats and carbohydrates in grams per day;

—the kinds of tobacco regularly consumed: light, dark, cigarettes, pipes, cigars or cigarillos;

—the kinds of alcoholic drinks: beer, wine, spirits.

The age at which the first signs of cancer of the pancreas appeared was determined by medical practitioners as the age of onset of the first symptoms. Possible errors were of little importance in view of the high mortality rate in the year following diagnosis.

*Statistical method*

The effects of the variables studied and of their interactions on the relative risk of pancreatic cancer were investigated by conditional logistic regression models. These models were introduced by Cox (1972) and adapted to retrospective case-control studies by Prentice & Breslow (1978). They were well suited for multivariate analysis of the relative risk.

Let $\mathbf{Z} = (Z_1, \ldots, Z_p)$ a vector. Each component $Z_i$ represents the value of a factor studied for a subject (cancer or control) or the interaction between several of these factors. If there is an interaction between two or more variables their combined effect on the logarithm of the relative risk is superior or inferior to the sum of the effects of each of them considered alone. The Log-relative risk for this subject, relatively to another with $\mathbf{Z} = \mathbf{Z}^{(0)}$ is expressed according to the regression model:

$$\text{Log RR} = (\mathbf{Z} - \mathbf{Z}^{(0)}).\boldsymbol{\beta}^t = \sum_{i=1}^{p} (Z_i - Z_i^{(0)}.\beta_i)$$

where:

RR is the relative risk, $p$ is the number of factors and interactions considered. Each component $\beta_i$ of the vector $\boldsymbol{\beta}$, quantifies the role of a factor (or an interaction) in the Log-relative risk variations. $\hat{\boldsymbol{\beta}}$ has to be estimated from the samples and we set $\hat{\boldsymbol{\beta}}$ its estimation. The estimation process is carried out by a conditional maximum likelihood method (Prentice & Breslow, 1978). Briefly, one can show that, if $\mathbf{Z}^{(1)}$, $\mathbf{Z}^{(2)}, \ldots, \mathbf{Z}^{(q)}$ are the exposure vectors of cancers and $\mathbf{Z}^{(q+1)}, \ldots, \mathbf{Z}^{(q+p)}$ those of controls, the conditional probability of observing $\mathbf{Z}^{(1)}, \ldots, \mathbf{Z}^{(q)}$ as cancer exposure vectors, $\mathbf{Z}^{(1)} \ldots \mathbf{Z}^{(p+q)}$ given is equal to:

$$\text{Exp}\left(\sum_{i=1}^{q} \mathbf{Z}^{(i)}.\boldsymbol{\beta}^t\right) \Big/ \sum_{I \varepsilon R} \text{Exp}\left(\sum_{i=1}^{q} [\mathbf{Z}^{(I_i)}\boldsymbol{\beta}^t]\right)$$

where $R$ is an index set comprising all the combinations of $q$ objects taken in $p+q$ or here all ways of selecting $p$ vectors among $p+q$ (Lubin, 1981).

If several strata have to be considered, the conditional likelihood is the product of terms as above on all the strata. An estimation of $\boldsymbol{\beta}$ is obtained by maximizing this function.

Asymptotic standard error $\hat{\sigma}_i$ for each $\beta_i$ component estimate is obtained from the information matrix (Cox, 1972) evaluated at the $\hat{\boldsymbol{\beta}}$ *estimate. Under* $\beta_i = 0$ hypothesis, the $\hat{\beta}_i/\hat{\sigma}_i$ (normal deviate) follows approximately a standard normal distribution and it is thus possible to test the significance of $\beta_i$ against zero. Moreover, if two conditional logistic regression models differ by the fact that one assumes $r$ coefficients $\beta_i$ equal to zero and the other not, the conditional ratio likelihood Chi square statistic (Cox, 1972; Prentice & Breslow, 1978) with $r$ degrees of freedom (d.f.) allows the testing of the contributions of $r$ factors (variables or interactions) to the variations of the relative risk of cancer of the pancreas. This makes it possible to perform a forward stepwise selection procedure to include in the model (at each step) the variable carrying the most information on relative risk variations, taking into account its relationships with the variables already included. Though different this method can be compared with a classical regression method, the dependent variable here being the Log-relative risk. However, the estimation process is quite different. Conditional maximum likelihood estimation allows the simultaneous inclusion of binary and continuous variables.

In the present study cancers and controls were stratified by age and sex. One or several cases were thus associated with one or several controls with the same sex and with the same age ($\pm 2.5$ y). This can be considered as an m–n design matching (Lubin, 1981). Conditional likelihood is then the product of the conditional likelihoods on the all strata. The resulting estimate of the relative risk as a function of the variables and their interactions allows the analysis of the variations of this risk with one factor, the others held constant.

Statistical computing was performed with the PECAN computer program of LUBIN written in Fortran ANS.

*Analytical protocol*

Cases and controls were distributed in 16 strata according to sex and 8 age classes.

The numbers of cancer patients and controls were different in each stratum (Table I). A dummy subject was taken as a reference to calculate the relative risk. The values of the variables were chosen for this dummy subject as the sample medians for fat, protein, carbohydrate and energy intakes and zero for the others: alcohol, tobacco and consumption durations. For this subject, the risk is thus set at unity. For continuous variables (fats, carbohydrates, alcohol...) we have tested a linear relationship between the Log-relative risk and each variable and also tested quadratic relationships. Indeed the Log-relative risk does not necessarily vary at the same rate in function of the values of a variable. For this purpose squared variables were included in the model.

### Results

The sample medians of the two groups are given in Table II with the sample means and s.d. for continuous variables. The mean age of cancer patients was $64.9 \pm 11.2$ y (females) and $64.2 \pm 10.2$ y (males). The variables considered are not independent. Fats, proteins and carbohydrates are interrelated. Moreover, dietary habits are modified by alcohol consumption. We emphasize that the statistical method for investigation of the Log-relative risk variations allows these interrelationships to be taken into account to some extent.

Each variable was first studied alone, then a more general model was designed. Table III shows the results obtained in the first case. Protein intake had no apparent effect on relative risk, but fat, carbohydrate and energy intakes had highly significant effects. It should be noted that the relative risk increases with fats ($\beta_i > 0$) and decreases with carbohydrate and energy intakes ($\beta_i < 0$). Mean daily alcohol consumption has a highly significant effect though duration has not. The same was true for tobacco but mean daily consumption had a smaller effect.

Spirits and aperitifs were not associated with a modification of the relative risk, but wine of high alcohol content had a positive effect and wine of low content had a negative effect. Beer consumption was of borderline significance. The kinds of tobacco (light or dark) had apparently no role. Because alcohol and tobacco were known to be correlated, we have included these variables in the same logistic conditional model (Table IV). The effect of tobacco was then not significant, but the duration of alcohol consumption now had a significant negative effect ($\beta_i < 0$).

Moreover, the inclusion of an interaction between alcohol and tobacco carried no information on the

**Table I** Definition of the strata: number of patients and controls in each stratum

| | *Males* | | *Females* | |
|---|---|---|---|---|
| *Age group (years)* | *Patients* | *Controls* | *Patients* | *Controls* |
| <45 | 1 | 4 | 3 | 5 |
| 45–49 | 3 | 5 | 1 | 3 |
| 50–54 | 3 | 10 | 1 | 7 |
| 55–59 | 4 | 13 | 7 | 14 |
| 60–64 | 10 | 18 | 3 | 19 |
| 65–69 | 5 | 23 | 6 | 21 |
| 70–74 | 6 | 13 | 5 | 16 |
| ≧75 | 5 | 14 | 6 | 14 |
| Totals | 37 | 100 | 32 | 99 |

**Table II** Means, standard deviations and medians for patients and controls

| | Controls | | Patients | |
|---|---|---|---|---|
| | Females | Males | Females | Males |
| | 65.2(a) | 64.9 | 64.9 | 64.2 |
| 1. Age | 10.6(b) | 10.08 | 11.2 | 10.2 |
| (y) | 65.0(c) | 65.0 | 67.0 | 64.0 |
| | 75.7 | 87.9 | 78.2 | 89.7 |
| | 9.0 | 12.7 | 26.0 | 21.0 |
| 2. Proteins (d) | 75.0 | 87.0 | 70.0 | 90.0 |
| | 69.1 | 82.3 | 87.6 | 96.2 |
| | 11.2 | 13.2 | 33.7 | 20.3 |
| 3. Fats (d) | 70.0 | 80.0 | 80.0 | 90.0 |
| | 301.7 | 330.4 | 240.2 | 283.6 |
| 4. Carbo- | 41.5 | 50.2 | 65.5 | 78.1 |
| hydrates (d) | 300.0 | 328.0 | 240.0 | 270.0 |
| | 19.3(82) | 43.0(97) | 47.7(25) | 64.0(35) |
| 5. Alcohol | 9.7 | 21.5 | 46.3 | 35.8 |
| intake (d) | 17.0 | 40.0 | 19.0 | 54.0 |
| 6. Alcohol | 43.4(82) | 46.1(97) | 42.8(25) | 45.5(35) |
| duration | 11.0 | 11.2 | 18.6 | 12.6 |
| (y) | 41.0 | 46.0 | 41.0 | 45.0 |
| | 6.4(34) | 16.4(82) | 20.4(14) | 19.9(28) |
| 7. Tobacco | 5.5 | 11.4 | 10.9 | 10.5 |
| intake (d) | 0 | 10.0 | 0 | 14.0 |
| 8. Tobacco | 32.1(34) | 42.4(82) | 36.1(14) | 41.8(28) |
| duration | 14.6 | 11.3 | 14.3 | 13.2 |
| (y) | 0 | 39.0 | 0 | 34.0 |

*For means and s.d. relative to tobacco and alcohol, calculations were performed only on the drinkers and smokers. Their numbers are given in parentheses. (a) mean. (b) s.d. (c) median. (d) intake in grams per day.

relative risk variations. Finally we have constructed a logistic conditional regression model by a forward stepwise manner.

At the first step the best variable alone, according to the conditional likelihood ratio statistic (constancy of the relative risk as a reference) was included. This variable was carbohydrate intake. At the following steps, according to the same criterion, another variable was included, thus taking into account its "relationships" with those already included. The variables (or factors) selected at each step are given in Table V. The variables were included in the model in the following order: carbohydrates, fats, mean daily alcohol, carbohydrates squared and duration of alcohol consumption.

The relative risk is negatively associated with carbohydrates and duration of alcohol consumption. The estimated regression equation for the Log-relative risk can be written:

$$\text{Log RR} = 0.079\ (f-77) - 31.10^{-4}\ (c-310) + 18\times 10^{-7}\ (c-310)^2 + 0.0215a - 0.033\ da$$

where "$f$", "$c$", "$a$" are set for fats, carbohydrates, alcohol in grams per day and "$da$" for duration of alcohol consumption in years.

Therefore consuming 10 g per day of fats in excess of the median (87 g) is associated with an augmentation of Log-relative risk equal to:

$$0.079\times(87-77) = 0.79$$

and the relative risk is multiplied by exp (0.79) = 2.21 relative to the dummy subject taken as a reference (Table VI). In this example the other variables are assumed to be constant.

**Table III** Logistic conditional regression model—Each variable alone

| *Variables* | $\hat{\beta}_i$ | *Normal deviate†* $\hat{\beta}_i/\hat{\sigma}_i$ | *R.R.‡* | *95% Confidence interval* *Lower limit* | *Upper limit* |
|---|---|---|---|---|---|
| Fats* | 0.060 | 5.45 | 1.80 (10 g/d) | 1.47 | 2.21 |
| Proteins* | 0.019 | 0.94 | 1.10 (10 g/d) | 0.90 | 1.32 |
| Carbohydrates* | −0.002 | −5.70 | 0.80 (100 g/d) | 0.76 | 0.88 |
| Energy* | −0.002 | −4.72 | 0.21 ($10^3$ g/d) | 0.59 | 0.31 |
| Alcohol duration (y) | −0.012 | −1.36 | 0.88 (10 y) | 0.75 | 1.05 |
| Alcohol intake* | 0.025 | 4.26 | 1.28 (10 g/d) | 1.14 | 1.44 |
| Tobacco duration (y) | 0.013 | 0.17 | 1.01 (10 y) | 0.25 | 5.10 |
| Tobacco intake* | 0.032 | 2.40 | 1.38 (10 g/d) | 1.06 | 1.79 |
| Beer (yes or no) | 0.65 | 1.91 | 1.92 | 0.98 | 3.73 |
| Aperitives (yes or no) | −0.680 | −1.86 | 0.50 | 0.25 | 1.04 |
| Spirits (yes or no) | −0.31 | −0.91 | 0.73 | 0.38 | 1.43 |
| Light tobacco (yes or no) | 0.28 | 0.76 | 1.32 | 0.64 | 2.72 |
| Dark tobacco (yes or no) | −0.31 | −0.86 | 0.73 | 0.36 | 1.43 |
| Cigars (yes or no) | 1.01 | 1.69 | 2.75 | 0.85 | 8.86 |
| Cigarettes (yes or no) | −0.49 | −1.34 | 0.61 | 0.30 | 1.25 |

*Intake in grams per day (g/d).
†Normal deviate: ratio of $\hat{\beta}_i$ at its "asymptotic" standard error $\hat{\sigma}_1$: for testing against $\beta_i = 0$, has to be compared with a standardized normal variable (significance for values superior at 1.96, or inferior at −1.96, $P = 0.05$).
‡R.R.: Relative risk.

**Table IV** Logistic conditional regression model for alcohol and tobacco only

| *Variables* | $\hat{\beta}_i$ | *Normal deviate* | *Relative risk is multiplied by* |
|---|---|---|---|
| alcohol intake | 0.29 | 4.38 | 1.34 (for 10 g/d) |
| alcohol duration | −0.275 | −2.68 | 0.76 (1 year) |
| tobacco intake | 0.24 | 1.40 | 1.26 (10 g/d) |
| tobacco duration | −0.13 | −1.37 | 0.87 (1 year) |

Conditional maximum likelihood −108.78.

Normal deviate has to be compared with a standardized normal variable (see legend Table III).

**Table V** Logistic conditional regression model forward selection of the variables

| *Step* | *Variable included* | *Maximum likelihood* | *Maximum likelihood ratio chi-square statistic** |
|---|---|---|---|
| 0 | none | −125.25 | |
| 1 | carbohydrates | −100.52 | 49.46 |
| 2 | fats | −72.99 | 55.06 |
| 3 | $(\text{carbohydrates})^2$ | −58.57 | 28.84 |
| 4 | alcohol intake | −55.27 | 6.60 |
| 5 | alcohol duration | −53.08 | 4.38 |

*Maximum likelihood ratio chi square statistic has to be compared with the value of a chi square variable with 1 degree of freedom. Amelioration of the fit between the model and the data is significantly different from zero, if its value is $>3.84$ ($P = 0.05$).

**Table VI** Logistic conditional regression model. Final model for log-relative risk*.

| *Variables* | $\hat{\beta}_i$ | *Normal deviate*† | *R.R.*‡ | 95% *Confidence interval* *Lower limit* | *Upper limit* |
|---|---|---|---|---|---|
| Carbohydrates (g/d) | $-31.10^{-4}$ | −5.61 | 0.73 | 0.66 | 0.82 |
| Fats intake (g/d) | 0.08 | 5.21 | 2.21 | 1.64 | 2.97 |
| Carbohydrates squared $(g/d)^2$ | $18.10^{-7}$ | 4.85 | 1.02 | 1.01 | 1.03 |
| Alcohol intake (g/d) | 0.02 | 2.61 | 1.24 | 1.05 | 1.44 |
| Alcohol duration (y) | −0.03 | −2.10 | 0.72 | 0.53 | 0.98 |

*Numbers are rounded.

†Normal deviate (see Table III).

‡R.R.: relative risk. It was calculated for 10 g/d over the median for fats, 100 g/d over the median for carbohydrates, 10 g/d over zero for alcohol intake and 10 y over zero for alcohol duration.

In the same way an alcohol consumption of 10 g per day is associated with an increase of Log-relative risk equal to 0.2. The relative risk is multiplied by 1.24 for each 10 g per day of alcohol consumption (Table VI).

For carbohydrate intake, other variables held constant, the Log-relative risk is decreased by 0.738 for each 100 g per day.

The estimations given for the Log-relative-risk are point estimations. Ninety-five per cent confidence intervals are thus given in Tables III and IV for the Log relative risk.

The relative risk calculated for an increment of each variable is displayed in Table VI. It is possible to plot the estimated Log-relative risk in function of each variable, the others held constant. The figure gives the variations of the Log-relative risk as a function of fat and alcohol consumption. This figure is only descriptive, the estimated values of the relative risk can vary around their point estimations. Ninety-five per cent confidence intervals (Table VI) give some information on this variation.

**Figure** Variations of the relative risk of cancer of the pancreas as a function of fat intake: (1) alcohol intake, 40 g/d; (2) 80 g/d; (3) 120 g/d. Duration of alcohol consumption = 30 y.

### Discussion

Investigation by case-control study of the association of the relative risk of cancer of the pancreas with dietary habits, tobacco and alcohol is open to criticism. In particular, the questions asked in the present survey concerned the subject's past consumptions. The responses may have been modified by the residence of the subjects (at home or in residential retirement homes for the controls) or by the disease itself, the cancer patients possibly responding differently from the controls. Consideration of the two samples (cases and controls) as independent can also introduce bias in spite of the *a posteriori* stratification. In particular this does not take into account the usual dwelling of the subjects. Moreover, only diet, alcohol and tobacco consumptions were taken into consideration. Other factors can interfere or play a role in cancer of the pancreas. For example, the common use of chemical products or metals in industry (e.g. naphthylamine, benzidine, aluminium ...) could affect the disease. Moreover cancer of the pancreas was frequently found in association with diabetes (Wynder *et al.*, 1973) and some hormonal factors (Lin & Kessler, 1981; Soloway & Sommers, 1966). Coffee consumption, a highly suspected factor, was not included in our study. Coffee is certainly related to smoking and drinking (and perhaps to dietary) habits. A confounding effect is thus possible with coffee. However, it seems improbable that the major findings of this study concerning fats and carbohydrates could be modified by its inclusion. Further investigations on this point are, however, necessary. The periods considered for determination of past habits can also introduce bias (Rothman, 1981).

These factors make it necessary to consider only the greatest effects in estimation of the relative risk. Stratification is a very useful method to avoid bias but is not always sufficient. External consideration can be very useful in the investigation of the roles of the factors retained. Analysis of the effect of each variable alone on the relative risk can lead to erroneous conclusions. The validity of the dietary questioning, used here, has already been tested in previous studies (Cubeau & Pequignot, 1976) and estimated consumptions can be considered reliable.

This study has shown that a high fat intake was associated with an increased relative risk of cancer. Previous studies based on total combined data from 19 countries have shown that fats are positively correlated to the National death rate from cancer of the pancreas (Segi *et al.*, 1969). Fats could contribute to carcinogenesis by increasing secretion of the pancreas. In particular, it has been shown in rats that a diet rich in unsaturated fats increased the induction of cancers by Azazerine (Roebuck, 1981). A high carbohydrate consumption is associated with a diminution of the relative risk. That is apparent whether analysis is performed on each variable or on several variables. It is possible that an augmentation of fat intake could be related to a reduction of carbohydrate intake. Since the cancer patients had a high fat consumption the lower carbohydrate intake could be a logical consequence of this uniform reduction rather than the result of some kind of protective effect exerted by carbohydrates. However, carbohydrate intake has a very highly significant effect in the present study and another hypothesis could be proposed. A diet with a higher fat/carbohydrate ratio (energy held constant) could imply a higher metabolism of fats and consequently exert its carcinogenic effect by the production of one or more fat metabolites.

Considered separately, alcohol and tobacco appear associated with an increased relative risk. However, in a multifactorial model including both, alcohol seems to be the predominant factor. Moreover, no interaction between them could be displayed. Several previous studies on this topic are in disagreement, some considering tobacco only, some alcohol only. However Allan & Imrie (1981) studying the prevalence of pancreatic and gastric cancers in Western Scotland where the incidence of lung cancer and heart disease is among the highest in the World, (this being undoubtedly related to the high incidence of heavy cigarette smoking), found a relatively low incidence of pancreatic cancer. These authors concluded that the incidence of pancreatic cancer is surprisingly low in Western Scotland if pancreatic cancer is related to smoking.

More recently, Lin *et al.* (1981) found no relation between pancreatic cancer and tobacco. The same authors showed that a consumption of wine greater than two glasses a day, increased the risk of pancreatic cancer in men. Burch & Ansari (1968) also suggested that there could exist a possible association between chronic alcoholism and carcinoma of the pancreas. In a large prospective study of British male doctors, Doll & Peto (1976) found only a slight excess of deaths from carcinoma of the pancreas among cigarette smokers (statistically not significant). Moolgavkar & Stevens (1981) found a positive association between cancer of the pancreas and smoking considered alone; they emphasized the need to take into consideration the cumulative consumption. The duration of tobacco consumption included in our model is not significant when duration and mean daily consumption of alcohol are included. In favour of the role of alcohol our study shows, surprisingly, that the relative risk is higher when the duration is shorter, the mean daily consumption being constant. This finding could mean that cancer patients are more sensitive to alcohol toxicity than controls and consequently their duration of alcohol consumption is shorter for the same daily intake of alcohol. The cancer patients probably ceased drinking before the onset of symptoms.

Moreover, this study showed the difficulties in determining the risk factors for cancer of the pancreas from a case-control survey design when the variables considered were interrelated. The analysis has to be carried out with a multifactorial model taking into account the interrelationships between the variables. The significant effect of one variable, considered alone, can be misleading.

The strength of the association between alcohol and tobacco does not allow these factors to be studied alone. The increase of risk with a factor is not a sufficient condition to attribute an aetiological role to a toxic agent. If alcohol is a direct or indirect risk factor for pancreatic cancer the underlying mechanism by which alcohol could act is not known. Repeated episodes of chronic inflammation in the pancreas could be involved; the daily alcohol intake of the chronic alcoholic could persistently stimulate glandular activity in the pancreas over many months or years and in turn predispose to carcinogenesis. Moreover, although the effect was not significant, beer consumption is associated with an increased relative risk (Table III) about twice that found in non-beer drinkers. This suggests that alcohol may not be directly involved but that the effect could be due to other contents of alcoholic beverages. The incidence of pancreatic cancer is higher in northern France (6.40–7.56 per $10^5$ inhabitants), where beer consumption is high, than in southern France where wine is the usual drink (4–4.60 per $10^5$ inhabitants). The amounts of nitrosamines in beer (and whisky) is higher than in wine and cognac. In our samples, 38/199 normal subjects were beer drinkers compared with 22/69 cancer patients. It should be noted also that the death rate from pancreatic cancer (Segi *et al.*, 1969) is higher in many countries where beer consumption is large such as Finland, Ireland, Britain, Denmark and Norway than in France, Italy and Spain where the national drink is wine. However, that is not true for Germany and Belgium.

It could be that both alcohol and the other agents present in alcoholic beverages are involved. It may be possible to discard tobacco in favour of alcohol as a risk factor, but in our study, this could be due also to the difference between light and dark tobacco, which latter is usually smoked in France. Indeed, the sugar content and smoke characteristics of these tobaccos are quite different. In particular, light tobacco smoke is acid and dark tobacco smoke alkaline. This alkalinity implies the presence of nicotine and pyrimide derivatives in the free state, thought to be less toxic as well as leading to less inhalation of dark tobacco smoke. Whether or not this is so, our study and the preceding considerations suggest that alcohol is a major aetiological factor for pancreatic cancer in France. The role of tobacco should be reinvestigated in a greater number of subjects.

The authors are grateful to: Prof. H. Lubin, National Cancer Institute, Bethesda, Maryland, U.S.A. for providing PECAN computer program; A. Roqueplo, dietitian, for the collection of data. They also wish to thank SEITA (The French State Tobacco Concern) for supplying information and Profs. R. Camatte, J. Delmont, A. Gauthier, A. Marquier & R. Michotey for their contributions. This study was supported by the "Groupement des Entreprises Françaises dans la Lutte contre le cancer" (GEFLUC) and "Association pour le développement de la recherche sur le cancer" (ADRC), INSERM ATP n° 47.76.77.

**References**

ALLAN, A. & IMRIE, C.W. (1981). The prevalence of pancreatic and gastric cancers in a population of heavy cigarette smokers. *Natl Cancer Project*, **6**, 3.

AUDIGIER, J.C., EUVRARD, P. & TUYNS, A.S. *et al.* (1976). Mortalité par cancer du pancréas en France. *Arch. Fr. Mal. App. Dig.*, **65**, 107.

BURCH, G.E. & ANSARI, A. (1968). Chronic alcoholism and carcinoma of the pancreas. *Arch. Intern. Med.*, **122**, 273.

COX, D.R. (1972). Regression models and life tables. *J.R. Statist. Soc. B.*, **34**, 187.

CUBEAU, J. & PEQUIGNOT, G. (1976). Enquête méthodolique testant la validité d'un interrogatoire portant sur l'alimentation passée d'un groupe de sujets du sexe masculin. *Rev. Epidem. et Santé Publ.*, **24**, 61.

CUKLE, H.S. & KINLEN, L.J. (1981). Coffee and cancer of the pancreas. *Br. J. Cancer*, **44**, 760.

DOLL, R. & PETO, R. (1976). Cancers in relation to smoking. Twenty years observation on male British doctors. *Br. Med. J.*, **ii**, 1525.

HIRAYAMA, T. (1975). Epidemiology of cancer of the stomach in the special reference to its recent decrease in Japan. *Cancer Res.*, **35**, 3460.

KRAIN, L.S. (1972). Cancer incidence. The crossing of the curves for stomach and pancreatic cancer. *Digestion*, **6**, 356.

LEVIN, D.L., CONNELLY, R.R. & DEVERSA, S.S. (1981). Demographic characteristics of cancer of the pancreas: mortality, incidence and survival. *Cancer*, **47**, 1456.

LIN, R.S. & KESSLER, I.I. (1981). A multifactorial model for pancreatic cancer in man. Epidemiologic evidence. *J. Am. Med. Ass.*, **245**, 147.

LUBIN, J.H. (1981). A computer program for the analysis of matched case-control studies. *Comput. Biomed. Res.*, **14**, 138.

MACMAHON, B., YEN, S., TRICHOPOULOS, D., WAREN, K. & MARDI, G. (1981). Coffee and cancer of the pancreas. *N. Engl. J. Med.*, **304**, 630.

MOOLGAVKAR, S.H. & STEVENS, R.G. (1981). Smoking and cancers of bladder and pancreas. Risks and temporal trends. *J. Natl Cancer Inst.*, **67**, 15.

MORGAN, R.G.H. & WORMSLEY, K.G. (1977). Cancer of the pancreas. *Gut*, **18**, 580.

PRENTICE, R.L. & BRESLOW, N.E. (1978). Retrospective studies and failure time models. *Biometrika*, **65**, 153.

ROEBUCK, B.D. (1981). Promotion by unsaturated fat of azazerine induced pancreatic carcinogenesis in the rat. *Cancer Res.*, **41**, 3961.

ROTHMAN, K.J. (1981). Induction and latent periods. *Am. J. Epidemiol.*, **114**, 253.

SEGI, M., KURIMARA, M. & MATSUYAMA, T. (1969). Cancer mortality for selected sites in 24 countries. n°5 (1964–1965). *Department of Public Health. Tokohu Univ. School of Medicine, Sendaï.*

SEIDMAN, H. (1970). Cancer death rates by site and sex for religious and socio economic groups in New York city. *Environ. Res.*, **3**, 234.

SOLOWAY, H.B. & SOMMERS, S.C. (1966). Endocrinopathy associated with pancreatic carcinomas: review of fast factors including hyperplasia and gonadotropic activity. *Ann. Surg.*, **164**, 300.

WYNDER, E.L., MABUCHI, K., MARUCHIN, N. & FORTNER, J.G. (1973). A case-control study of cancer of the pancreas. *Cancer*, **31**, 641.